# Genomic landscape of *bla*_NDM-1_- and *bla*_OXA-181_-carrying *Citrobacter portucalensis* sequence type 151 strains from hospital wastewater in Ghana

**DOI:** 10.1128/aem.02108-25

**Published:** 2026-03-31

**Authors:** Frederick Ofosu Appiah, Yusuke Ota, Jennifer Seyram Amedior, Emmanuel Darko, Mitsunori Yoshida, Masato Suzuki, Yoshihiko Hoshino, Toshihiko Suzuki, Tomoko Ishino, Anthony Ablordey, Ryoichi Saito

**Affiliations:** 1 Department of Molecular Microbiology and Immunology, Institute of Science Tokyo 13290, Tokyo, Japan; 2 Department of Parasitology and Tropical Medicine, Institute of Science Tokyo 13290, Tokyo, Japan; 3 Department of Bacteriology, Noguchi Memorial Institute for Medical Research, Accra, Ghana; 4 Department of Mycobacteriology, Leprosy Research Center, National Institute of Infectious Diseases 231182 , Tokyo, Japan; 5 Antimicrobial Resistance Research Center, National Institute of Infectious Diseases 13511, Tokyo, Japan; 6 Department of Bacterial Pathogenesis, Infection and Host Response, Institute of Science Tokyo 13290, Tokyo, Japan; University of Minnesota Twin Cities, St. Paul, Minnesota, USA

**Keywords:** *Citrobacter portucalensis*, hospital wastewater, *bla*_NDM-1_, *bla*_OXA-181_, IncX3, ColKP3

## Abstract

Carbapenemases represent one of the major driving forces of antimicrobial resistance (AMR), which has been recognized as a significant public health concern. Additionally, hospital wastewater (HWW) serves as a critical hotspot for the spread of antibiotic-resistant bacteria (ARB) and genes (ARGs). In this study, we report the isolation of difficult-to-identify *Citrobacter portucalensis* strains that harbor *bla*_NDM-1_ and *bla*_OXA-181_ from hospital wastewater in Ghana. Wastewater was collected at the Effia Nkwanta Regional Hospital of Ghana, and carbapenemase producers were screened using selective media and multiplex PCR. Antimicrobial susceptibility testing, conjugation, and whole-genome sequencing were performed on the isolates. Two strains (HW7-CARBA and HW8-CARBA) isolated from hospital wastewater were initially identified as *Citrobacter freundii* but confirmed as *C. portucalensis* by whole-genome sequencing. These strains were assigned to ST151 and harbored ARGs consistent with their multidrug-resistant phenotype. The *bla*_NDM-1_ gene was located on the ~45.0 kb IncX3 plasmid (pHW7-CARBA-NDM-1), which was transferable in the conjugation assay, while the *bla*_OXA-181_ gene was bracketed with IS*26* on the ~90.0 kb nonconjugative ColKP3 plasmid (pHW8-CARBA-OXA-181). pHW7-CARBA-NDM-1 showed close phylogenetic similarity to a plasmid from China, whereas pHW8-CARBA-OXA-181 was closely related to the *bla*_OXA-181_ plasmid derived from *Escherichia coli* isolated from the stool sample of a pediatric patient with diarrhea in Ghana. Consequently, HWW serves as a hotspot for ARB and ARGs, increasing the risk of AMR proliferation. Addressing these challenges is essential for protecting public health and contributing to global efforts to preserve antibiotic efficacy. Recommendations include implementing comprehensive AMR-focused regulatory frameworks.

**IMPORTANCE:**

The operations of hospitals generate wastewater that serves as a critical hotspot for antibiotic-resistant bacteria and genes. Many hospitals discharge untreated wastewater, leading to the widespread occurrence of carbapenemase-producing *Enterobacteriaceae*, which presents significant challenges for environmental health. The significance of our research lies in determining the genomic landscape of antibiotic-resistant genes in the less-explored *Citrobacter portucalensis* isolated from hospital wastewater. This research will contribute to preventing the spread of environmental resistance, implementing antimicrobial resistance monitoring standards, and protecting vulnerable populations from exposure.

## INTRODUCTION

The emergence and prevalence of antimicrobial resistance (AMR) are increasingly recognized as significant public health concerns on a global scale (1). Bacterial AMR is reported to result in approximately 1.14 million deaths annually, with the majority occurring in low- and middle-income countries (LMICs) (2). In contrast, the United States and Europe record approximately 23,000 and 25,000 deaths each year, respectively (3). Unfortunately, the environmental contribution to this pressing issue is often underestimated, despite its potential as a key driver (4). Currently, there are no documented figures that accurately reflect the environmental contribution to the overall AMR health crisis.

Hospitals are vital for advancing medical science and supporting healthcare systems (5), but they also face significant challenges regarding environmental health due to the wastewater they produce (6, 7). Hospital wastewater (HWW) is particularly concerning as it contains antibiotic-resistant bacteria (ARB) and genes (ARGs), toxic chemicals, antibiotic residues, and pathogens (6, 7). The discharge of wastewater without proper treatment is particularly common in LMICs, leading to the dissemination of ARGs and ARB (8–10). Alarmingly, some farmers use untreated HWW to irrigate crops and vegetables meant for human consumption, thereby directly exposing individuals to ARGs and ARB (11). Moreover, community wastewater is relatively less toxic than HWW due to lower contaminant levels (12, 13), positioning hospitals as critical hotspots for AMR and environmental pollution.

Furthermore, carbapenemases are the most significant enzymes that neutralize antibiotics, especially carbapenems (14). The majority of acquired carbapenemases belong to three of the four known classes of β-lactamases: Ambler class A enzymes, such as *Klebsiella pneumoniae* carbapenemase (KPC) types; Ambler class B enzymes, or metallo-β-lactamases, including Verona integron-encoded metallo-β-lactamase (VIM), imipenemase (IMP), and New Delhi metallo-β-lactamase (NDM) types; and Ambler class D enzymes, or oxacillinases, such as OXA-23, OXA-48, and OXA-181 types (15, 16). NDM is a type of metallo-β-lactamases that can break down the majority of β-lactam antibiotics, which are typically considered first-line drugs to treat infections (17, 18). The emergence of NDM-producing bacteria significantly limits clinical therapeutic options, complicating medical interventions for infectious diseases caused by these pathogens (19, 20). NDM-1 was first described in 2008 in India, and since that time, *Enterobacteriaceae* possessing NDM-1 have been isolated from patients in various countries, including the USA, Canada, the United Kingdom, Europe, Australia, Oman, Japan, Singapore, and Africa (19–21). In Ghana, multiple reports of *bla*_NDM-1_ have been documented (22–24).

Since the discovery of Ambler class D enzymes OXA-48, a total of 48 variants have been identified (https://www.ncbi.nlm.nih.gov/pathogens/refgene/#oxa-48, accessed on 20 April 2025), with OXA-181 being the second-most common (25). The spread of OXA-181 producers has been documented in France (26), the Netherlands (27), and India (28). Additionally, the dissemination of the *bla*_OXA-181_ gene has been reported in Africa (29–31), with specific occurrences noted in Ghana (32–34).

Finally, *Citrobacter portucalensis* is challenging to identify and is often underestimated (35); however, carbapenemase-producing strains have been documented. The first report of *bla*_NDM-1_ producing *C. portucalensis* was documented in China, marking the first clinical case of *bla*_NDM-1_-carrying *C. portucalensis* (35). This was subsequently followed by several reports, including a drug-resistant clinical strain harboring both *bla*_KPC-2_ and *bla*_NDM-1_ (36), as well as an extensively drug-resistant clinical *C. portucalensis* that exhibited the rare coexistence of *bla*_SFO-1_, *bla*_KPC-2_, and *bla*_NDM-1_ genes (37).

We hereby report, for the first time, the isolation of two multidrug-resistant *C. portucalensis* strains harboring *bla*_NDM-1_ and *bla*_OXA-181_ from hospital wastewater in Ghana.

## RESULTS

### Bacterial identification, antimicrobial susceptibility testing, and conjugal transfer of *bla*_NDM-1_ and *bla*_OXA-181_

Two strains (HW7-CARBA and HW8-CARBA) were obtained from hospital wastewater and initially identified as *C. freundii* by 16S rRNA gene sequencing but confirmed as *C. portucalensis* with an average nucleotide identity (ANI) score 98.51% using strain A60 (GCF_002042885.1) as the reference. HW7-CARBA and HW8-CARBA exhibited resistance or intermediate resistance to all classes of β-lactam antibiotics, levofloxacin, and gentamicin, with the exception of HW8-CARBA, which demonstrated sensitivity to imipenem (Table 1). Additionally, both strains were sensitive to amikacin, tigecycline, and colistin (Table 1). The HW7-CARBA and HW8-CARBA strains also tested positive for modified carbapenemase inactivation (mCIM) and harbored the *bla*_NDM-1_ and *bla*_OXA-181_ genes, respectively, and the ETEST analysis also confirmed the resistance of both strains to meropenem. Conjugation analysis revealed that HW7-CARBA successfully transferred the *bla*_NDM-1_gene to the recipient strain *Escherichia coli* J53, while the *bla*_OXA-181_ gene from HW8-CARBA was not transferred. PCR testing confirmed the presence of *bla*_NDM-1_ (Fig. S1), and 16S rRNA sequencing identified the transconjugant strain as *E. coli*. The transconjugant Tc-NDM-1 also conferred resistance to β-lactam antibiotics, with the exception of aztreonam, and demonstrated sensitivity to amikacin, gentamicin, tigecycline, and colistin (Table 1). The oriTfinder results indicated that the conjugative region of pHW7-CARBA-NDM-1 contained all four modules: the origin of transfer site (*oriT*), relaxase gene, gene encoding type IV coupling protein (T4CP), and gene cluster for the bacterial type IV secretion system (T4SS). In contrast, pHW8-CARBA-OXA-181 contained only the relaxase gene. Both strains harbored resistant genes for heavy metals such as mercury (*merA, merD, merE, merT,* and *merR*) and arsenic (*arsA, arsB, arsC, arsD,* and *arsR*) (Tables S4 and S5).

**TABLE 1:** Antimicrobial susceptibility profiles of the two isolates and a transconjugant^*a*^

Antimicrobial agent	MIC (μg/mL) for:		
	HW7-CARBA	Tc-NDM-1	HW8-CARBA
Ampicillin	>16 (R)	>16 (R)	>16 (R)
Ampicillin/sulbactam	>16/8 (R)	>16/8 (R)	>16/8 (R)
Cefazolin	>16 (R)	>16 (R)	>16 (R)
Ceftazidime	>8 (R)	>8 (R)	>8 (R)
Cefpodoxime	>4 (R)	>4 (R)	>4 (R)
Ceftriaxone	>32 (R)	>32 (R)	>32 (R)
Cefepime	>16 (R)	>16 (R)	>16 (R)
Levofloxacin	>1 (R)	>1 (R)	>1 (R)
Imipenem	2 (l)	2 (l)	1 (S)
Meropenem	4 (R)	2 (I)	2 (I)
Amikacin	≤8 (S)	≤8 (S)	≤16 (S)
Gentamicin	>8 (R)	2 (S)	>8 (R)
Aztreonam	>8 (R)	2 (S)	>8 (R)
Tigecycline	≤1 (S)	0.25 (S)	≤1 (S)
Colistin	≤1 (S)	≤1 (S)	≤1 (S)

^*a*^ S, susceptible; R, resistant; l, intermediate; Tc-NDM-1, transconjugant.

### Genomic characterization of HW7-CARBA and HW8-CARBA and phylogeny analysis

The genome of HW7-CARBA comprised a chromosome of 5,012,851 bp and 7 circular plasmids ranging in size from 3,361 bp to 241,933 bp (Table 2). This strain harbored multiple ARGs, including those for β-lactams (*bla*_NDM-1_, *bla*_OXA-1_, *bla*_CTX-M-15_, *bla*_TEM-1B,_ and *bla*_CMY-124_), tetracycline (*tet(D*)), aminoglycosides (*aac(6′)-Ib-cr*,), quinolone (*qnrS1 and qnrB76*), and sulfonamide/dihydrofolate reductase inhibitors (*sul1*, *sul2*, and *dfrA12*) (Table 2). Notably, the *bla*_NDM-1_ gene was localized on a 44,962 bp IncX3 plasmid. In comparison, the genome of HW8-CARBA consisted of a chromosome of 5,233,847 bp and 6 circular plasmids ranging from 3,361 bp to 239,022 bp (Table 2). HW8-CARBA also contained ARGs for β-lactams (*bla*_OXA-181_, *bla*_OXA-1_, *bla*_CTX-M-15_, *bla*_TEM-1B,_ and *bla*_CMY-124_), tetracycline (*tet(D*)), aminoglycosides (*aac(6′)-Ib-cr*,), quinolone (*qnrS1 and qnrB76*), and sulfonamide/dihydrofolate reductase inhibitors (*sul1*, *sul2*, and *dfrA12*) (Table 2). The *bla*_OXA-181_ gene was found on an 89,673 bp ColKP3-type plasmid.

**TABLE 2:** Chromosome and plasmid features of HW7-CARBA and HW8-CARBA

Strain name	Chromosome/ plasmid	Size (bp)	MLST	Plasmid incompatibility group	Antibiotic resistance gene(s)
HW7-CARBA	Chromosome	5,012,851	ST151	–^*a*^	*bla*_CMY-124_, *qnrB76*
	pHW7-CARBA-1	241,933		–	*bla*_OXA-1_, *bla*_TEM-1B_, *tet(D), sul1, sul2, dfrA12, aac(3)-lld, aadA2, aac(6’)-lb-cr), mph(A), catB3, cattA2*
	pHW7-CARBA-2	85,230		IncFII (CF)	*qnrS1*
	pHW7-CARBA-3	72,754		repB (R1701)	*mph(A), bla_TEM-1B_, bla_CTX-M-15_ aph(6)-ld, aph(6)-ld, aac(3)-lld, aac(3)-lld*)
	pHW7-CARBA-4	55,112		–	–
	pHW7-CARBA-NDM-1	44,962		IncX	*bla*_NDM-1_
	pHW7-CARBA-6	3,482		–	–
	pHW7-CARBA-7	3,361		–	–
HW8-CARBA	Chromosome	5,233,847	ST151	–	*bla*_CMY-124_, *bla*_TEM-1B,_*bla*_OXA-1_, *qnrB76, qnrB1*
	pHW8-CARBA-1	239,022		–	*aac(3)-lld, aadA2,aac(6’)-lb-cr, tet(D), sul1, dfrA12, mph(A), catB3, catA2*
	pHW8-CARBA-2	96,068		IncFII (CF)	–
	pHW8-CARBA-OXA-181	89,673		ColKP3	*aac(3)-lld, aac(3)-lla, aph(6)-ld, aph(6)-ld), bla*_CTX-M-15_, *bla*_TEM-1B_, *bla*_OXA-181_, *mph(A), qnrS1*
	pHW8-CARBA-4	9,264		IncQ1	*sul2*
	pHW8-CARBA-5	3,482		–	–
	pHW8-CARBA-6	3,361		–	–

^*a*^ – indicates the absence of plasmid incompatiblity group or ARGs.

**Fig 1:**
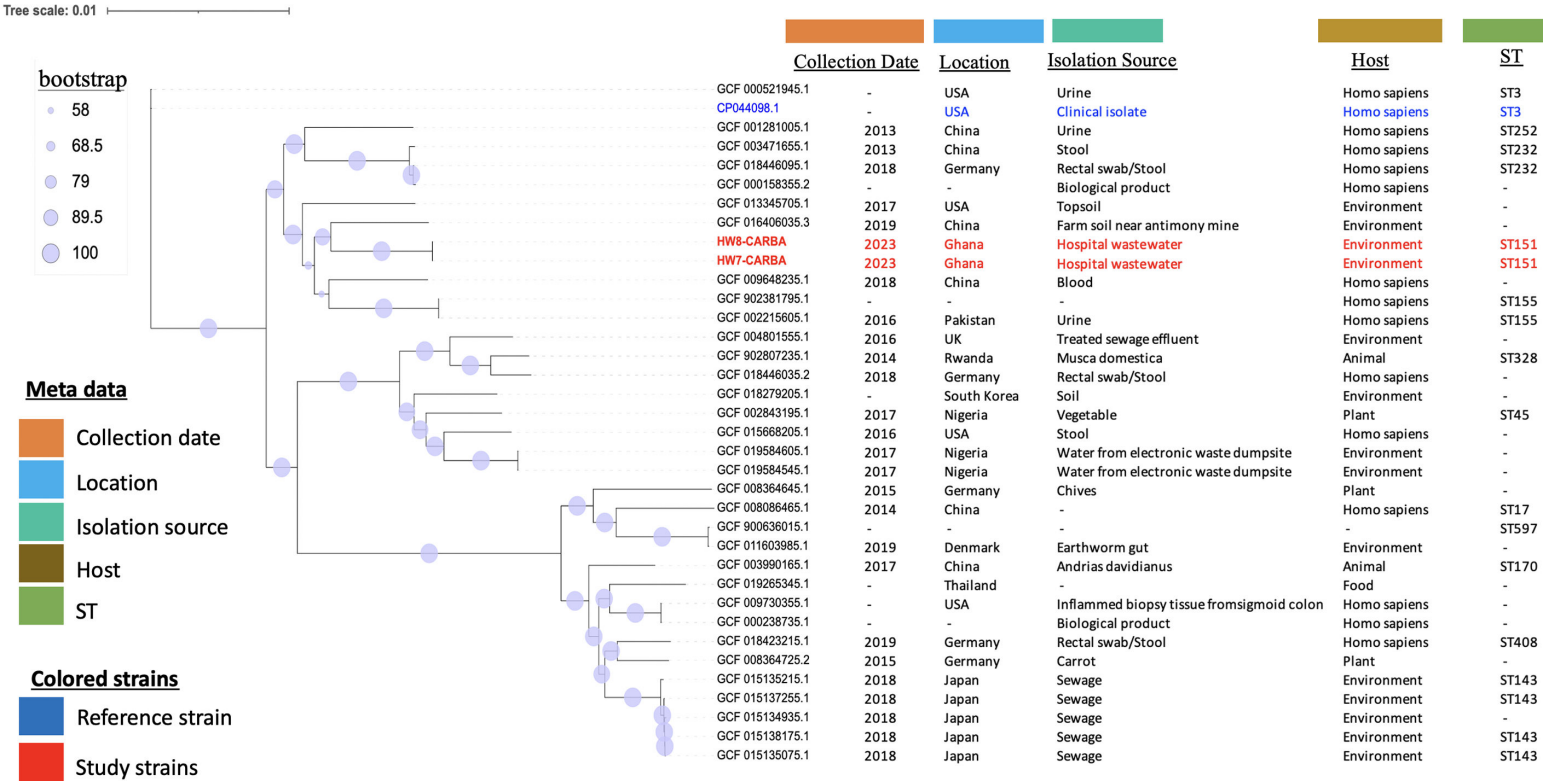
Population distribution of *C. portucalensis* strains (HW7-CARBA and HW8-CARBA) with 34 *C. portucalensis* genomes. The phylogenetic tree was generated using the maximum-likelihood approach. Bootstrap analysis was employed to assess the support level (expressed as a percentage) for each cluster of related taxa, indicated by blue dots adjacent to the branches. The scale of sequence divergence is represented by the accompanying bar. The colors used in the tree correspond to various factors, including the collection date, location, source of isolation, host, and ST of the strains. A dash (-) signifies that data are not available. The reference strain (CP044098) is represented in blue, while the strains from our study are shown in red.

The HW7-CARBA and HW8-CARBA strains were both assigned to sequence type (ST) 151. We investigated their phylogeny within the context of global *C. portucalensis* collections together with 34 *C. portucalensis* strains. The metadata for these strains are presented in Fig. 1, with the standard strain of *C. portucalensis* (CP044098) as the reference strain. The phylogenetic tree was divided into two primary groups, each containing distinct subclades, while the reference strain (CP044098) and an American strain (GCF_016406035) were out-grouped (Fig. 1). The HW7-CARBA and HW8-CARBA strains were classified within the same group, indicating that they shared similar homology; however, they did not exhibit ancestral homology with the standard strain CP044098 (Fig. 1). Furthermore, HW7-CARBA and HW8-CARBA exhibited similar homology to GCF_016406035, a *C. portucalensis* strain isolated from farm soil near an antimony mine in China in 2019 (Fig. 1). However, the ST of GCF_016406035 was not available.

**Fig 2:**
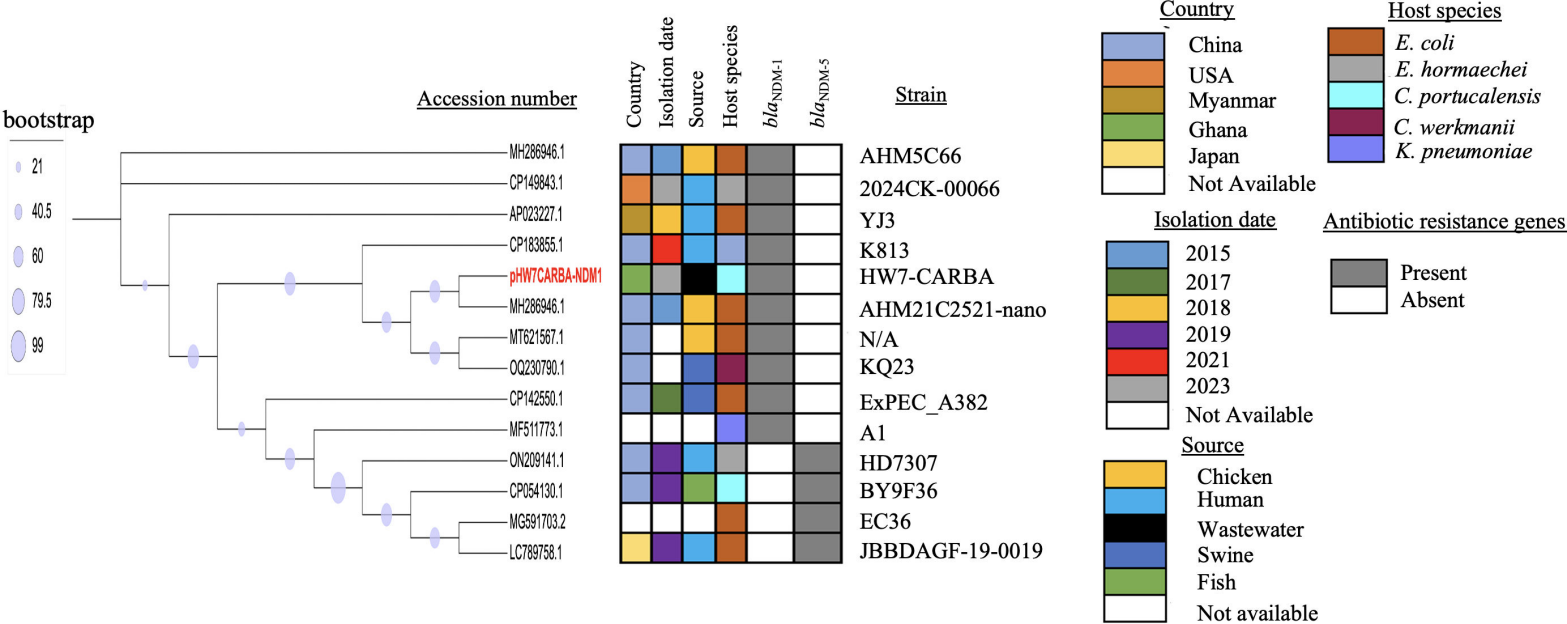
Analysis of the evolutionary relatedness of pHW7-CARBA-NDM-1 (indicated in red) and other *bla*_NDM-1_ and *bla*_NDM-5_ plasmids. Branch patterns were assessed using 1,000 bootstraps, with symbols and respective values indicated in the legend. The colors depicted correspond to the countries, isolation dates, sources, and host species of the plasmids. The ash color denotes the presence of genes, while the white color signifies their absence.

We determined the phylogenetic relatedness of the IncX3- *bla*_NDM-1_ plasmid from HW7-CARBA (pHW7-CARBA-NDM-1) and 13 other plasmids exhibiting high homology through BLAST analysis. The analysis indicated that pHW7-CARBA-NDM-1 shared a close genetic distance with a *bla*_NDM-1_ plasmid (MH286946.1) in *E. coli* recovered from chicken in China. Additionally, it shared a common lineage with two other *bla*_NDM-1_ plasmids (MT621567.1 and OQ230790.1) from China, hosted by *E. coli* and *C. werkmanii*, respectively, and recovered from chicken and swine (Fig. 2).

**Fig 3:**
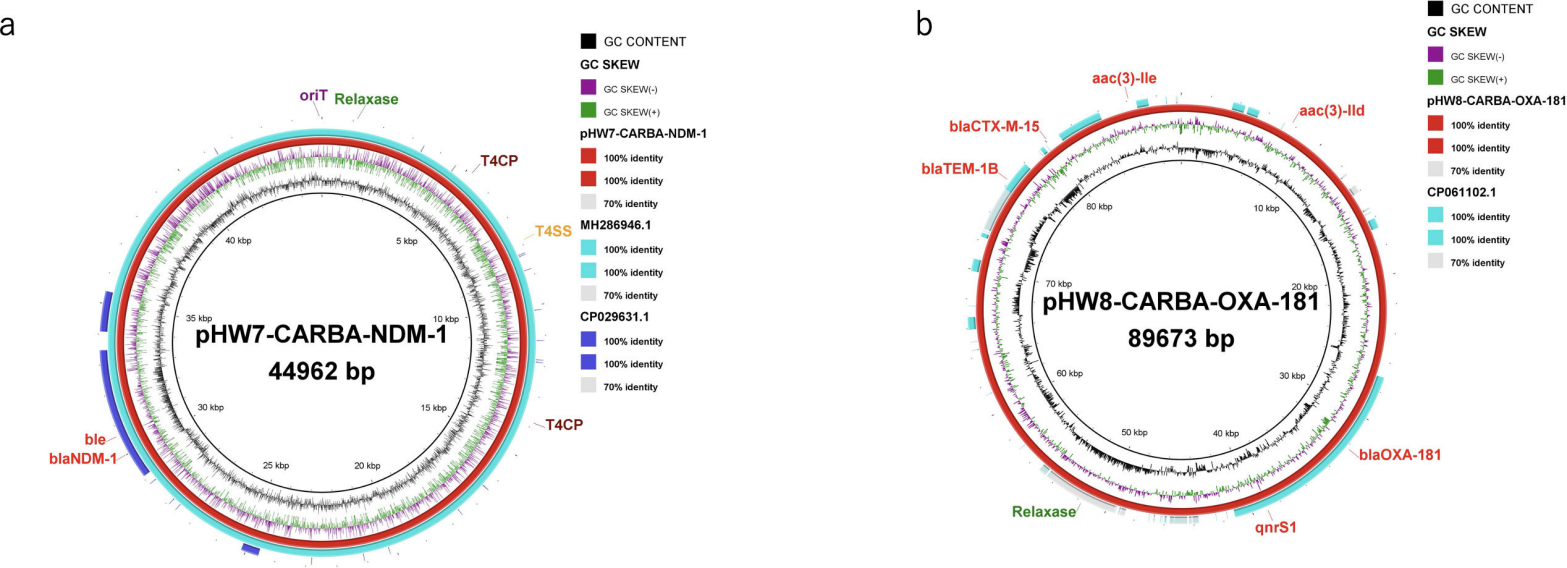
(**a**) A circular comparison of pHW7-CARBA-NDM-1 and two IncX3-*bla*_NDM-1_-containing plasmids (MH286946.1 and CP029631.1). The red, green, and blue colors represent pHW7-CARBA-NDM-1, MH286946.1, and CP029631.1, respectively. (**b**) A circular comparison of pHW8-CARBA-OXA-181 and CP061102.1, a *bla*_OXA-181_-containing plasmid. The red and green colors represent pHW8-CARBA-OXA-181 and CP061102.1, respectively. The homology between the plasmids is shown by the percentage identity in the figure legend.

We conducted a mapping of the contigs of pHW7-CARBA-NDM-1 to the sequences of MH286946.1 and CP029631.1, the latter being a *bla*_NDM-1_ plasmid isolated from a hospital in Ghana. The mapping results indicated that our plasmid was identical to MH286946.1 with 100% coverage, while it exhibited no similarity to CP029631.1, with only the *bla*_NDM-1_ and *ble* region showing 100% identity (Fig. 3a). The circular comparison revealed a large, conserved scaffold likely containing numerous conjugative genes, as previously reported (38).

**Fig 4:**
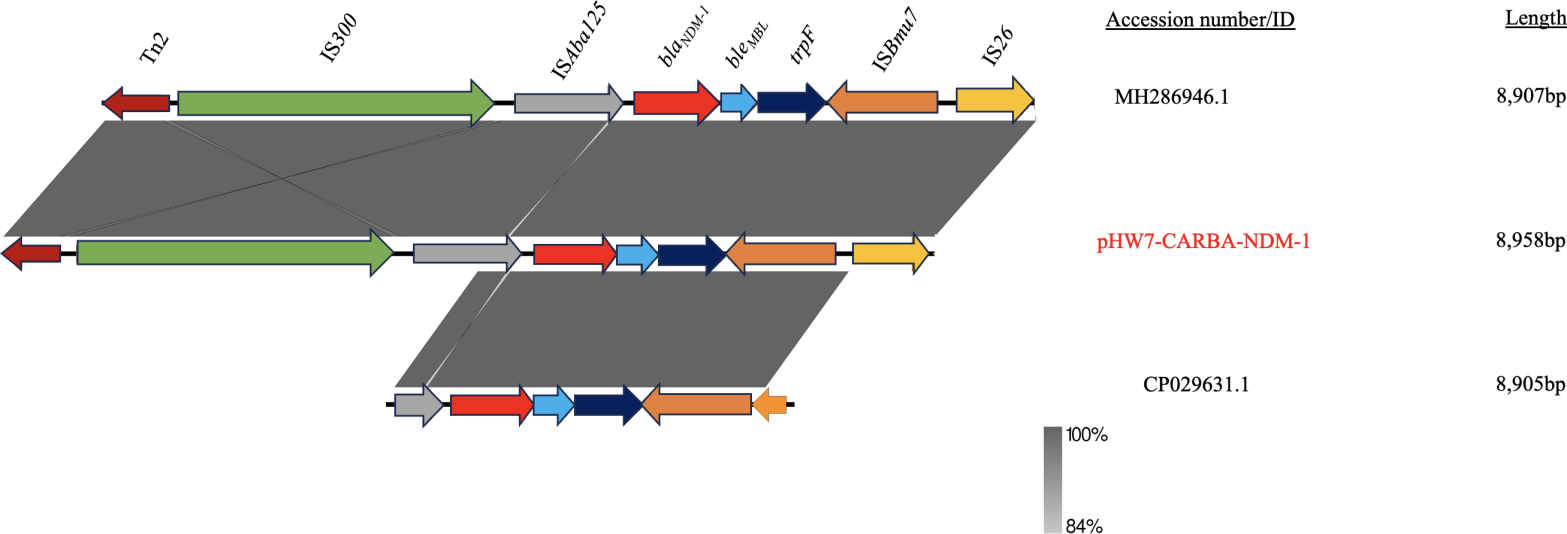
Comparison of the genetic environment of *bla*_NDM-1_. Arrows depict the genes and sequences upstream and downstream of *bla*_NDM-1_in their transcriptional directions, as detected by sequencing. The structure of MH286946.1 was reversed to enable easy comparison. Shaded regions indicate regions that are common to the plasmids.

A detailed examination of the genetic environment of *bla*_NDM-1_ on pHW7-CARBA-NDM-1 and MH286946.1 revealed a sequence of open reading frames (ORFs): *Tn2-*IS*300-*IS*Aba125-bla*_NDM-1_-*ble*_MBL_-*trpF*-IS*Bmu7*-IS*26*. The *bla*_NDM-1_ gene was flanked upstream by the insertion sequence IS*Aba125* and downstream by the bleomycin resistance gene *ble*_MBL_, both bordered by Tn2 upstream and IS*26* downstream. Conversely, CP029631.1 exhibited a similar sequence but contained a truncated IS*Aba125* and did not include Tn2 and IS*300* (Fig. 4). The large, shaded ash color denotes the similarities in the genetic environments of *bla*_NDM-1_ among the plasmids, while the white color signifies the differences (Fig. 4).

**Fig 5:**
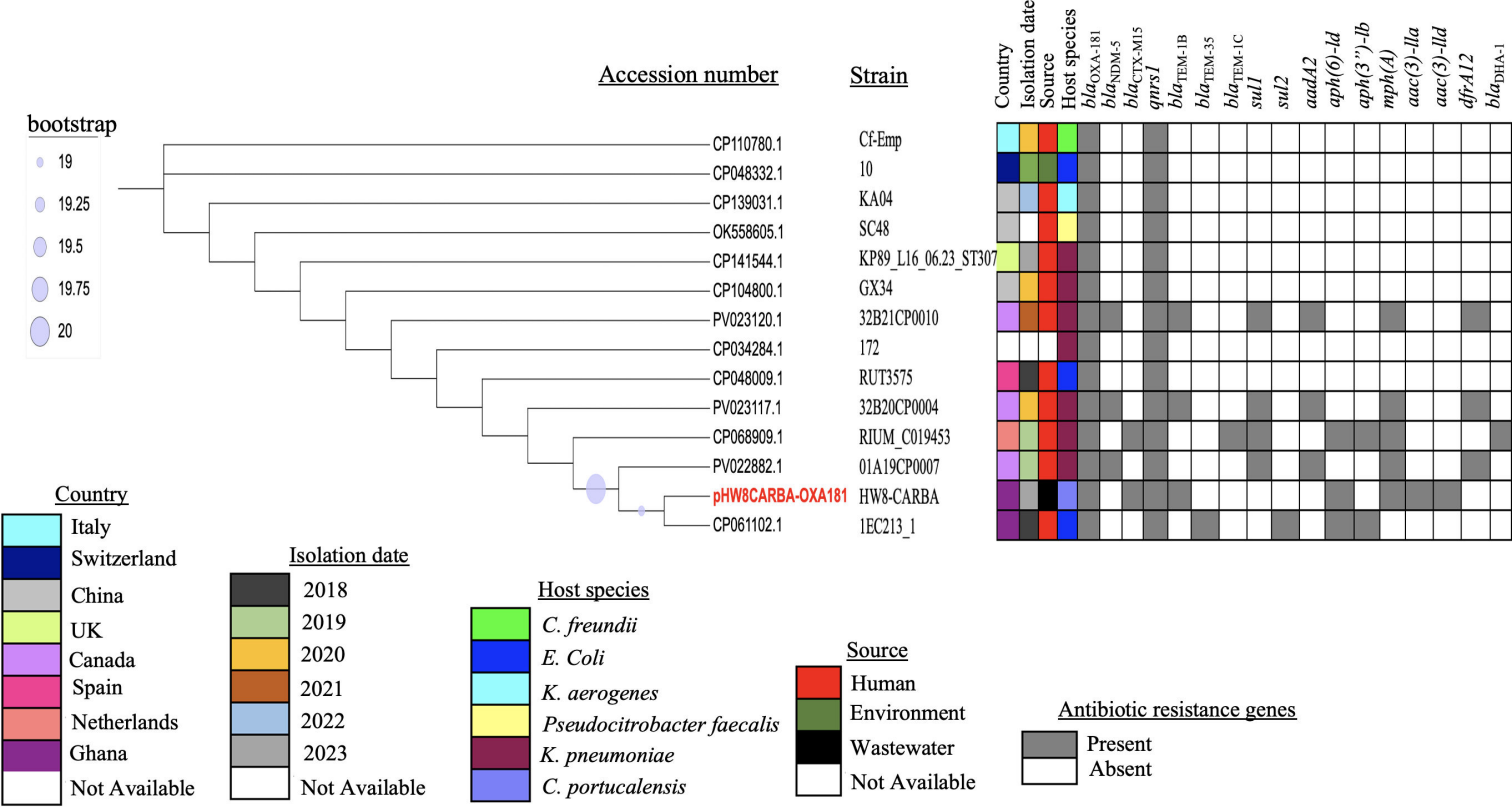
Evolutionary relatedness of pHW8-CARBA-OXA-181 (indicated in red) and other *bla*_OXA-181_ plasmids. Branch patterns were assessed using 1,000 bootstraps, with symbols and respective values indicated in the legend. The colors depicted correspond to the countries, isolation dates, sources, and host species of the plasmids. The ash color denotes the presence of genes, while the white color signifies their absence.

The evolutionary relatedness analysis revealed that pHW8-CARBA-OXA-181 shares a close genetic relationship with the *bla*_OXA-181_ plasmid (CP061102.1) derived from *E. coli*. Notably, plasmid CP061102.1 was isolated from the stool sample of a pediatric patient with diarrhea in the western region of Ghana (Fig. 5). Furthermore, the CP061102.1 plasmid contained several additional ARGs, including *qnrS1*, *bla*_TEM-35,_*sul2, aph(6)-ld, and aph(3”)-lb*. In comparison, pHW8-CARBA-OXA-181 harbored a different set of resistance genes, such as *bla*_CTX-M-15_, *qnrS1*, *bla*_TEM-1B_, *aph(6)-ld, mph(A), aac(3)-lla, and aac(3)-lld* (Fig. 5). Additionally, their AMR profiles exhibited phenotypic differences. The phylogenetic analysis involved the *bla*_OXA-181_ plasmid (pHW8-CARBA-OXA-181) and 13 other plasmids that exhibited high homology (Fig. 5).

The results from the NCBI BLAST analysis indicated that CP061102.1 shared a 99.99% identity and 31% query coverage with pHW8-CARBA-OXA-181. The BRIG comprehensive visual comparison also revealed structural differences between the two plasmids (Fig. 3b). The specific contig section of pHW8-CARBA-OXA-181 that exhibited a perfect 100% identity to CP061102.1 was the *bla*_OXA-181_ and *qnrS1* (Fig. 3b). Furthermore, a larger portion of the plasmid displayed less than 70% similarity or was completely absent, which could be attributed to deletions or the absence of a scaffold for conjugative genes in pHW8-CARBA-OXA-181 (Fig. 3b).

**Fig 6:**
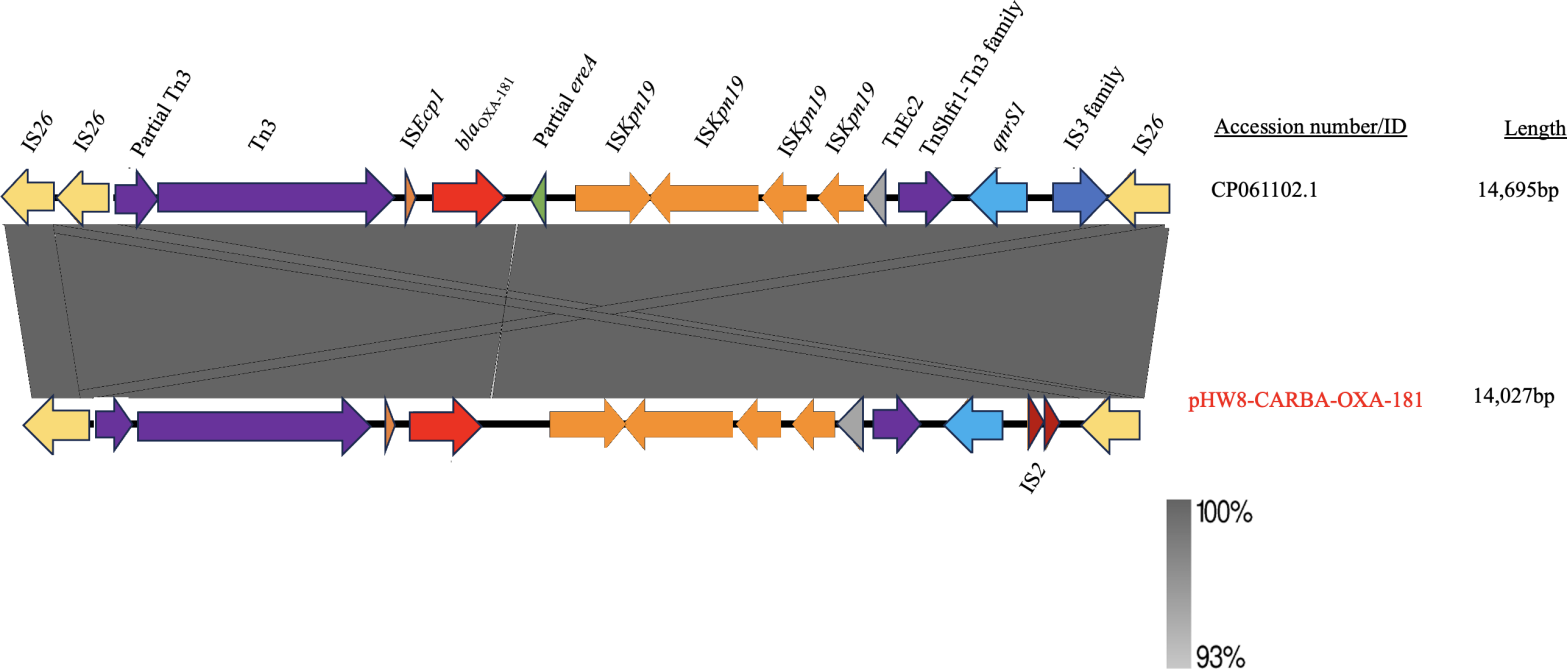
Comparison of the genetic environment of *bla*_OXA-181_. Arrows depict the genes and sequences upstream and downstream of *bla*_OXA-181_in their transcriptional directions, as detected by sequencing. Shaded regions indicate regions that are common to both plasmids.

The comparison of the genetic environment of *bla*_OXA-181_ between CP061102.1 and pHW8-CARBA-OXA-181 revealed notable similarities, with some open reading frames differing in presence or absence. The upstream region of *bla*_OXA-181_ on pHW8-CARBA-OXA-181 exhibited a sequence of IS*Ecp1*-Tn3-partial-Tn3-IS*26*, whereas the upstream region of *bla*_OXA-181_ on CP061102.1 showed IS*Ecp1-*Tn3-partial-Tn3-IS*26*-IS*26* (Fig. 6). Notably, CP061102.1 harbored two copies of IS26, whereas pHW8-CARBA-OXA-181 possessed only a single copy (Fig. 6). Comparatively, the downstream region of *bla*_OXA-181_ on CP061102.1 showcased a sequence of partial *ereA*-IS*Kpn19*-IS*Kpn19*-IS*Kpn19*-IS*Kpn19*-TnEc2-Tnshfr1-*qnrS1*-IS*3* family-IS*26*, while pHW8-CARBA-OXA-181 exhibited a sequence of IS*Kpn19*-IS*Kpn19*-IS*Kpn19*-IS*Kpn19*-TnEc2-Tnshfr1-*qnrS1*-IS2-IS*26* (Fig. 6). The immediate downstream region of *bla*_OXA-181_ on CP061102.1 harbored a partial *ereA* and IS*3* family, which were absent on pHW8-CARBA-OXA-181, which instead contained IS*2* (Fig. 6). However, both plasmids were bracketed by IS*26* in the upstream and downstream regions of *bla*_OXA-181_ (Fig. 6). The large, shaded ash color denotes the similarities in the genetic environments of *bla*_OXA-181_ between the plasmids (Fig. 6).

## DISCUSSION

*Citrobacter* spp., a genus of gram-negative coliform bacteria within the *Enterobacteriaceae* family, is found widely in the environment, including soil, water, and wastewater, as well as in the intestines of humans and animals (39). *Citrobacter* spp. possesses several virulence factors that pathogenic microorganisms use to express their pathogenicity and enhance survival in hosts (40). These factors are closely related to disease and can be elicited during antimicrobial therapy (40). Notable virulence factors include proteolysis, hemolysis, and biofilm formation, which have been previously observed (41). Studies have reported that the use of conventional tests and MALDI-TOF MS for differentiating species of *Citrobacter* to recognize clinically significant species has been challenging (42–44), thereby highlighting the need for more advanced techniques. Cao et al. reported that among 488 *C. freundii* genomes downloaded from the GenBank database, 55 were identified as *C. portucalensis*, with an ANI value greater than 95% (35). Three of these 55 misidentified strains were isolated from Africa. This misidentification corroborates the findings of this study, where our strains were initially identified as *C. freundii* but later confirmed as *C. portucalensis*. These strains harbored mercury (Hg)-resistant genes, which have been reported to co-occur with multiple ARGs in bacterial isolates from food animals, fish, and humans due to the ubiquitous presence of Hg in the environment (45–47). Hg has been extensively utilized as an antimicrobial agent in healthcare, agriculture, aquaculture, and industry (48). Therefore, it can be speculated that the survival of *C. portucalensis* strains against Hg in HWW is based on the Hg-resistant genes they possess. In fact, Li et al. have reported that it is important to reduce Hg concentrations in sewage to minimize the co-selection potential of ARGs in humans and animals (49). The relatively lesser-explored *C. portucalensis* is gradually gaining attention for its potential contributions to various fields. There have been multiple reports from both clinical and nonclinical samples since strain A60 was identified in 2017 as a novel species of *C. portucalensis* in Portugal (50). This includes strain MBTC-1222, which was isolated from vegetables in Nigeria in 2018 (51), and strain NR-12, isolated from poultry in Bangladesh in 2019 (52). In 2020, strain RIT669 was documented (53), followed by strain 3839 reported to be isolated from the sputum of a 72-year-old man with polydipsia, polyuria, and cough in China in 2021 (35). In 2022, strain K218 was reported (36), and in 2023, researchers isolated a *C. portucalensis* strain from an endangered marine animal that succumbed to sepsis (54). In 2024, Guo et al. reported a clinical case of *C. portucalensis* isolated from the feces of a 56-year-old man with acute diarrhea in China (37), and a strain was also isolated from edible snails in Nigeria (55). This rapidly emerging body of evidence underscores the increasing dissemination of *C. portucalensis* in both the environment and clinical domains, but there is a relative lack of exploration of this pathogen in Africa. Although reports have been documented in Nigeria, Egypt, and South Africa, there are no available data regarding *C. portucalensis* in Ghana. Given its diverse adaptability and the clinical underestimation resulting from misidentification, there is a necessity for ongoing molecular surveillance to mitigate the burden of AMR in accordance with the One Health concept.

The 34 *C. portucalensis* strains involved in the maximum-likelihood phylogenetic analysis were sourced from various locations, with the main collection sites being China, Germany, the United States, and Japan. The *C. portucalensis* FDAARGOS_617 reference strain (CP044098) out-grouped from the group containing HW7-CARBA and HW8-CARBA strains gives an indication that there is a less relevant evolutionary relationship. Furthermore, GCF_016406035 with an unknown ST type, isolated from farm soil near an antimony mine in China, showed similar homology to the strains in this study, both classified as ST151, a sequence type not yet documented in scientific literature. Numerous reports have described diverse STs of *C. portucalensis* globally, including ST170 and ST85 from hospitalized patients (36, 37), ST264 from marine animals that succumbed to sepsis (54), and ST881, ST882, ST985, and ST896 from edible snails (55). The classification of our study strains as ST151 may indicate unique characteristics or adaptations. Since this is the first report of ST151 *C. portucalensis* strains in Ghana, it is challenging to ascertain the possible transmission events and the close relatedness to the Chinese isolate. It can be speculated that the strains isolated from hospital wastewater may have originated from the feces of a patient who has traveled to China, or it is also possible that these strains were introduced directly from China, indicating potential pathways for the spread of these strains. This situation necessitates further research to elucidate the prevalence of this pathogen and its evolutionary routes in Ghana.

Since the initial report of *bla*_NDM-1_ -producing *C. portucalensis* in China (35), there have been several subsequent reports (36, 37, 53). There have been several reports of *bla*_NDM-1_-producing *Enterobacteriaceae* in Ghana and throughout Africa; however, there is a lack of documentation regarding *C. portucalensis* as a producer of this gene. The *bla*_NDM-1_ -producing *C. portucalensis* from this study also exhibited resistance to the carbapenem, as previously documented (36, 37, 53). An extensively resistant *bla*_NDM-1_ -producing strain of *C. portucalensis* was isolated from the bloody sputum of a 76-year-old male patient diagnosed with type 2 diabetes mellitus (35). Additionally, another study reported the isolation of an extensively resistant *bla*_NDM-1_ -producing *C. portucalensis* from the blood of a 56-year-old male patient admitted to the neurosurgery department. The patient was diagnosed with left thalamic hemorrhage, hydrocephalus, and hypertension (36). Also, the isolation of two multidrug-resistant *bla*_NDM-1_ -producing strains of *Klebsiella pneumoniae* from burn patients in Ghana has been documented (23). These observations underscore the clinical significance of monitoring such resistant strains within hospital settings. The intermediate resistance to meropenem and imipenem exhibited by the transconjugant strain indicates that the *bla*_NDM-1_ gene is solely responsible for the hydrolysis of carbapenems. Furthermore, the presence of additional ARGs in strain HW7-CARBA, apart from *bla*_NDM-1_, signifies the substantial level of resistance to various antibiotics and underscores the potential health risks associated with this strain. First identified in India in 2007 (36), OXA-181 has since been reported in various countries around the world, including Ghana, affecting humans, animals, and environmental samples (32, 33, 56–58). Despite its classification as a carbapenemase, OXA-181 exhibits instances of low imipenem and meropenem MIC values, which fall within the susceptible range according to the current clinical breakpoints established by the European Committee on Antimicrobial Susceptibility Testing (EUCAST) and Clinical & Laboratory Standards Institute (CLSI). This has been reported in previous studies (59, 60); however, our study strain HW8-CARBA showed resistance to meropenem while being sensitive to imipenem. The varying resistance levels observed in strains carrying *bla*_OXA-181_ toward meropenem raise critical questions about the underlying factors contributing to this diversity as some strains show resistance while others remain sensitive (61). This phenomenon raises important questions regarding the clinical management of infections caused by strains with this enzyme. Moreover, the low carbapenemase activity of OXA-48 enzymes may pose challenges in detecting OXA-181 carbapenemases in clinical settings, potentially leading to an underestimation of OXA-181-like-producing *Enterobacteriaceae* (62). Reports concerning *bla*_OXA-181_-producing *C. portucalensis* are infrequent, with only one documented case in Peru, where it was isolated from the bronchial secretions of a patient (63). Furthermore, the same study also reported the isolation of multidrug-resistant *bla*_OXA-181_-producing strains of *K. pneumoniae* and *E. coli* from the urine and blood of patients. Additionally, a study conducted in Ghana reported the isolation of two multidrug-resistant *bla*_OXA-181_-producing strains of *E. coli* from the stools of pediatric patients diagnosed with diarrhea (32). Ghana has encountered significant challenges related to AMR, resulting in 5,900 attributable deaths and an additional 25,300 associated deaths (64). The country was ranked 36th globally in terms of age-standardized mortality rates associated with AMR per 100,000 population (64). Donkor et al. reported that the primary determinants of AMR and its dissemination are the weak enforcement and non-adherence to practice standards, policies, and regulations governing the access to and utilization of antimicrobial agents in both human and veterinary medicine (65). Consequently, the isolation of multidrug-resistant *C. portucalensis* strains from HWW in Ghana informs the evolution of resistance patterns and underscores the necessity for implementing strategies to mitigate the risks posed by these strains. To the best of our knowledge, this is the first report of *bla*_NDM-1-_ and *bla*_OXA-181_-producing *C. portucalensis* in Ghana.

NDM-1 is often associated with IncX3-type plasmids (66), which consist of plasmids characterized by a narrow host range (67). The analysis presented in Fig. 2 indicated that 14 IncX3-type plasmids were isolated from 5 different host species. Notably, the presence of pHW7-CARBA-NDM-1 in *C. portucalensis*, which is closely related to MH286946.1 in *E. coli*, suggests that IncX3-*bla*_NDM-1_ plasmids have broadened their host preference. Therefore, understanding the driving forces behind this spread is essential. The genetic environment of *bla*_NDM-1_ on pHW7-CARBA-NDM-1 exhibits similarity to MH286946.1, but not to CP029631.1, a *bla*_NDM-1_ plasmid that we isolated in our previous research from the urine of a patient at a teaching hospital in Ghana. This observation reflects the diversity in the ORFs surrounding the gene, which may contribute to the resistance mechanisms associated with the *bla*_NDM-1_ gene against various antibiotics. The *ble*_MBL_ gene, which confers resistance to the antitumor glycopeptide bleomycin, was located downstream of the *bla*_NDM-1_ gene (68). Previous studies indicate that IS*Aba125* is typically involved in the formation of Tn125, a complex transposon associated with the plasmid-mediated spread of *bla*_NDM-1_ (69, 70). The location of *ble*_MBL_ and IS*Aba125* downstream and upstream of *bla*_NDM-1_, respectively, has been reported in previous studies (36). Mobile genetic elements (MGEs) such as Tn2 and IS*300* have been shown to play a significant role in the dissemination of *bla*_NDM-1_, with IS*26* being one of the primary contributors (71). Our findings also affirm the transferability of pHW7-CARBA-NDM-1, indicating the potential for strain HW7-CARBA to disseminate the *bla*_NDM-1_ gene among other *Enterobacteriaceae*, which poses a significant health risk.

Several plasmids carrying *bla*_OXA-181_ have been reported, including ColKP3, IncX3, IncT, and IncN1 replicons (72, 73). pHW8-CARBA-OXA-181 was found to be closely related to a ColKP3-type OXA-181 plasmid (CP061102.1) that we isolated in our previous research from an *E. coli* species in the stool of a pediatric patient with diarrhea from the same western region of Ghana. Although their phenotypic AMR profiles differ, it is speculated that the OXA-181 gene has been transmitted to other bacterial species. It has been reported that the conjugative transfer regions of self-transmissible MGEs typically comprise oriT, relaxase gene, T4CP, and T4SS (74). Consequently, the failure of plasmid pHW8-CARBA-OXA-181 to achieve conjugation may be attributed to the absence of a complete conjugation system. Nonetheless, the transfer of the *bla*_OXA-181_ gene through IS26-mediated co-integration with other plasmids is still possible. These findings highlight the need for future surveillance in this region from a One Health perspective, especially in animals. This is because the prevalence of *bla*_OXA-181_-producing *Enterobacteriaceae* has been reported in companion animals globally (75–77), yet there are no reports of such prevalence in Ghana, highlighting a potential gap in research or data collection in the region. We recommend that strategic educational policies should be developed and made readily accessible to communities, healthcare facilities, and food production sectors, providing timely and consolidated information on AMR and antimicrobial use. Furthermore, the hospital facilities should ensure proper sewage treatment to prevent indiscriminate spillage into neighboring communities. This information is essential for mitigating the adverse effects of AMR on human and animal health. It should also be recognized that environmental and food systems serve as vectors for the transmission of resistant pathogens. Moreover, continuous research involving clinical samples, animals, and environmental samples is necessary to monitor the spread of ARB and ARGs. IS*Ecp1* was found immediately upstream of *bla*_OXA-181_ on pHW8-CARBA-OXA-181 and CP061102.1, consistent with findings reported by Potron et al. and other β-lactamase resistance genes, such as *bla*_CTX-M_, *bla*_CMY_, and *bla*_ACC_ (78–81). IS*Ecp1* is likely involved in the expression of *bla*_OXA-181_ and serves as an efficient genetic vehicle for spreading clinically significant extended-spectrum β-lactamases (ESBLs), such as plasmid-encoded cephalosporinases and ESBLs (82); however, it was not found on plasmid CP061102.1. Reports indicate that *ereA* is located downstream of *bla*_OXA-181_ (83), as identified in CP061102.1; however, it may not have been annotated at the downstream region of *bla*_OXA-181_ on pHW8-CARBA-OXA-181 (Fig. 6). The *ereA* gene encodes erythromycin esterase type 1. Mobile genetic elements like IS*26*, IS*Ecp1*, and IS*2* are crucial for forming and propagating multi-resistant regions. IS*26* often carries antibiotic resistance genes and facilitates their spread through replicative transposition. It is known to mediate the formation of cointegrates between two DNA molecules, with the donor molecule containing IS*26* (84). pHW8-CARBA-OXA-181, in addition to *bla*_OXA-181_, also carried *bla*_CTX-M-15_ and *qnrS1,* which raises significant concerns about the risk of multidrug-resistant infections, complicating treatment strategies. Moreover, their resistance mechanisms may interact synergistically, reducing the effectiveness of these antibiotics and creating obstacles for successful patient outcomes.

## MATERIALS AND METHODS

### Sample collection site and bacterial isolation

In June 2023, a volume of 2,000 mL of wastewater was collected from the Effia Nkwanta Regional Hospital, located in Takoradi, Ghana. The wastewater underwent vigorous shaking, after which a ten-fold serial dilution was performed, and 100 µL was subsequently inoculated onto CHROagar mSuper CARBA agar plates. (Kanto Chemical Co., INC. Tokyo, Japan) and incubated at 37°C overnight. Subsequently, five to eight morphologically distinct colonies from each plate were passaged onto fresh agar plates and preserved in skim milk (Morinaga Milk, Tokyo, Japan) for subsequent analysis.

### Species identification by 16S rRNA gene sequencing

Crude DNA was extracted from the isolates using CICA Geneus DNA Extraction Reagent (Kanto Chemical Co., Tokyo, Japan), as described earlier (85). We prepared a 10 µL PCR reaction mix consisting of 5 µL of 2× Emerald premix (Takara Bio Inc., Shiga, Japan), 0.5 µL of each forward 8UA (3 µM, 5′-AGAGTTTGATCMTGGCTCAG-3′) and reverse primer 1485B (3 µM, 5′-TACGGTTACCTTGTTACGAC-3′), 3 µL of nuclease-free water, and 1 µL of the DNA template. The polymerase chain reaction (PCR) process began with an initial denaturation at 98°C for 1 min, followed by 30 cycles of 98°C for 5 s, 57°C for 10 s, and 72°C for 1 min, concluding with a final extension at 72°C for 3 min. After amplification, the PCR products were purified using EXOSAP IT (Applied Biosystems, Foster City, CA, USA) and sequenced on a 3730xl DNA Analyzer with the BigDye Terminator v3.1 Cycle Sequencing Kit (Applied Biosystems). Identification of the sequences was conducted through National Center for Biotechnology Information (NCBI) BLAST searches utilizing a threshold identity of 100% and an e-value cutoff of 0.05, in accordance with the results obtained from sequencing.

### Antimicrobial susceptibility testing and carbapenemase detection

The MICs of antibiotics were determined by broth micro-dilution using DP45 dry plates (Eiken Chemical Co., Tokyo, Japan) and MicroSCAN (Beckman Coulter, California, US), as described in the CLSI M100-S32 guideline (86). Isolates were further screened for carbapenemase production following the mCIM method, as outlined in the CLSI guidelines. The major carbapenemase genes, including those encoding VIM-, IMP-, NDM-, KPC-, and OXA-48-like carbapenemases, were screened (22).

### Conjugation assays

The transferability of the *bla*_NDM-1_ and *bla*_OXA-181_ genes was assessed using the agar mating conjugal method, following a modified protocol previously described (32, 87). The sodium azide-resistant *E. coli* strain J53 served as the recipient strain. The 1 mL of recipient and 1 mL of donor strains (HW7-CARBA and HW8CARBA) were mixed in a 1:1 ratio, and the bacterial suspension or mixture was centrifuged at 10,000 × *g* for 3 min. Afterward, 1,800 μL of the supernatant was discarded, and the remaining 200 μL of each mix was vortexed and inoculated onto tryptone soy agar plates (Oxoid Ltd, Basingstoke, UK). The plates were incubated at 37°C overnight, and the bacterial cells were collected into 1 mL of 0.9% sterile physiological saline after the incubation to prepare a 10-fold serial dilution. The dilutions were spread onto bromothymol blue lactose agar plates (Eiken Chemical Co.) supplemented with 2 μg/mL meropenem and 100 μg/mL sodium azide to isolate the transconjugants (Tc-NDM-1) (87). The 100 μg/mL sodium azide was used as a selection criterion for the recipients (87). The presence of the *bla*_NDM-1_-containing plasmid was verified using PCR for carbapenemase genes and species identification.

### Genome sequencing and analysis

Genomic DNA was extracted using the Magattract HMW DNA kit (Qiagen, Hildon, Germany) following the manufacturer’s guidelines. For short-read sequencing, libraries were constructed with the Illumina DNA prep method, utilizing the IDT for Illumina DNA/RNA UD Indexes, and sequenced on the Illumina Miniseq (Illumina Inc, San Diego, USA), resulting in reads of 150 bp in length (88). The quality of the short reads was evaluated using FastQC v0.11.9 (https://github.com/s-andrews/FastQC, accessed on 20 July 2024) (88). Reads were subsequently trimmed and filtered using fastp v0.23.1 (https://doi.org/10.1093/bioinformatics/bty560, accessed on 20 July 2024) (88). For long-read sequencing, libraries were prepared according to the manufacturer’s instructions using the native barcoding and ligation sequencing kits EXP-NB104 and SQK-LSK114 (Oxford Nanopore Technologies) (88). The libraries were then loaded onto MinION flow cells (R10.4.1), and sequencing was performed on the MinION Mk 1B (Oxford Nanopore Technologies) (88). The barcoded Fast5 reads generated were basecalled using Guppy v1.1.4. (https://community.nanoporetech.com/protocols/Guppy-protocol/ [accessed on 15 September 2024]) (88). Demultiplexing of reads and adapter sequence trimming were performed using Porechop v0.2.4 (89). Long reads with low quality (MinION Q < 10) and long reads ≤1,000 bp were filtered out with filtlong (https://github.com/rrwick/Filtlong [accessed on 15 September 2024]). Hybrid assembly of long read and short reads was done with Unicycler v0.4.8 (90). Identification of isolates was confirmed by whole-genome sequencing with Genome Taxonomy Database Toolkit (GTDB-Tk-v1.7.0) (91). Genomes were uploaded into the RAST online server for annotation (92). Assembled genomes were screened for acquired ARGs, STs, and replicon types through the web-based center for genomic epidemiology databases, ResFinder v4.7.2 (93), MLST (94), and PlasmidFinder (95). oriTfinder (96) was used to identify the origin of transfers in the plasmids that harbored *bla*_NDM-1_ and *bla*_OXA-181._ Comprehensive visual comparison of the plasmid sequences was performed using the BLAST Ring Image Generator (BRIG) v.0.98 (97). ARGs were also identified through the web-based database using the resistance gene identifier (RGI) (98). Virulence factors, ARGs, and resistant genes for heavy metals were identified using AMRfinderPlus v4.0.19 (99). Genetic context and comparison of the identified *bla*_NDM-1_ and *bla*_OXA-181_ gene structures were done using Easy Fig v.2.2.2 (100).

### Phylogenetic analysis

To investigate the strains within a global context, a maximum-likelihood phylogenetic tree was constructed using a total of 34 *C. portucalensis* strains from the NCBI GenBank database (accessed on 17 June 2025), with assembly levels classified as scaffold, chromosome, or complete (Table S1) (18), including our study strains (HW7-CARBA and HW8-CARBA). Additionally, the phylogeny of pHW7-CARBA-NDM-1 and pHW8-CARBA-OXA-181 was evaluated, along with their respective 13 *bla*_NDM-1_ and *bla*_OXA-181_ plasmids, which exhibit high homology (percentage identity >99%). The plasmids were analyzed using NCBI BLASTn (accessed on 25 June 2025) with default settings, and selections were made from the first list of results, with a preference for plasmids derived from *C. portucalensis* and also isolated from Ghana or Africa. The raw reads were assembled using Unicycler v0.4.8 (90), and the genomes were annotated with Prokka v1.13 (101). Pangenome analysis was conducted utilizing Roary v3:12.0 (102). The plasmid phylogeny was based on core genes, and the resultant core-genome alignment file from Roary was utilized in Iqtree v2.2.0.3 to generate a phylogenetic tree with 1,000 bootstrapping replicates (103). The visualization and annotation of the trees were executed using ITOL v7 (https://itol.embl.de/ [accessed on 20 June 2025]), as previously described (104). The list of *C. portucalensis* genomes, *bla*_OXA-181,_ and *bla*_NDM-1_ plasmid sequences is summarized in Tables S1 to S3, respectively.

### Conclusion

This study represents the first instance of the identification of NDM-1- and OXA-181-producing *C. portucalensis* from HWW in Ghana. The resistant genes *bla*_NDM-1_ and *bla*_OXA-181_ were found to be associated with IncX3-type and ColKP3-type plasmids, respectively, suggesting the potential for horizontal transfer and further dissemination of these resistance genes among *Enterobacteriaceae* and other sources. Consequently, it is crucial to maintain continuous monitoring and ongoing attention to the detection of the difficult-to-identify *C. portucalensis* carrying *bla*_NDM-1_, *bla*_OXA-181_, and other resistance genes.

## Data Availability

The data sets presented in this study can be found in the NCBI BioProject database under accession number PRJNA473419. The BioSample accession numbers of HW7-CARBA and HW8-CARBA are SAMN54532435 (JBVFOY000000000) and SAMN50771552 (JBQSUO000000000), respectively.

## SUPPLEMENTAL MATERIAL

Fig. S1 aem.02108-25-s0001.docx PCR confirmation of *bla*_NDM-1_ in eight selected transconjugants.

Supplemental tables aem.02108-25-s0002.xlsx Tables S1 to S9.

ASM does not own the copyrights to Supplemental Material that may be linked to, or accessed through, an article. The authors have granted ASM a non-exclusive, world-wide license to publish the Supplemental Material files. Please contact the corresponding author directly for reuse.
