# Supplementary material for: Genomic landscape of blaNDM-1- and blaOXA-181-carrying Citrobacter portucalensis sequence type 151 strains from hospital wastewater in Ghana
Source: Appl Environ Microbiol. 2026 Mar 31;92(4):e02108-25. doi: 10.1128/aem.02108-25 (PMC13101484; doi:10.1128/aem.02108-25)
Supplement: Fig. S1 — PCR confirmation of blaNDM-1 in eight selected transconjugants. [file aem.02108-25-s0001.docx]

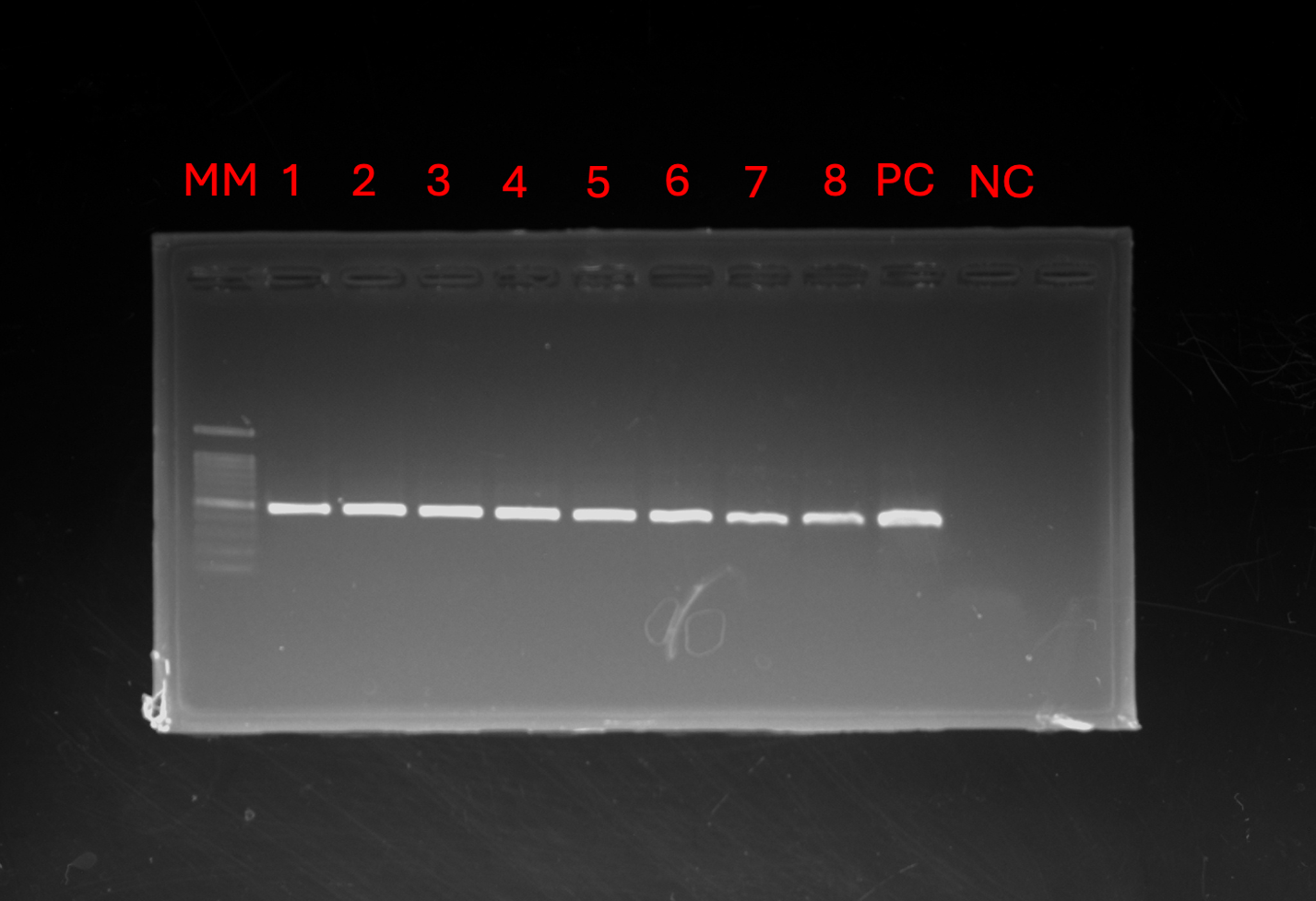


Figure S1: PCR confirmation of *bla*_NDM-1_ in eight selected transconjugants. The MM, PC and NC denote 100bp molecular marker, positive control and negative control respectively.
